# Reversible Congenital Hypogonadotropic Hypogonadism in Patients with *CHD7*, *FGFR1* or *GNRHR* Mutations

**DOI:** 10.1371/journal.pone.0039450

**Published:** 2012-06-19

**Authors:** Eeva-Maria Laitinen, Johanna Tommiska, Timo Sane, Kirsi Vaaralahti, Jorma Toppari, Taneli Raivio

**Affiliations:** 1 Children's Hospital, Helsinki University Central Hospital, Helsinki, Finland; 2 Institute of Biomedicine/Physiology, Biomedicum Helsinki, University of Helsinki, Helsinki, Finland; 3 Division of Endocrinology, Department of Medicine, Helsinki University Central Hospital, Helsinki, Finland; 4 Department of Physiology, University of Turku, Turku, Finland; 5 Department of Pediatrics, University of Turku, Turku, Finland; University of Muenster, Germany

## Abstract

**Background:**

Congenital hypogonadotropic hypogonadism (HH) is a rare cause for delayed or absent puberty. These patients may recover from HH spontaneously in adulthood. To date, it is not possible to predict who will undergo HH reversal later in life. Herein we investigated whether Finnish patients with reversal of congenital hypogonadotropic hypogonadism (HH) have common phenotypic or genotypic features.

**Methods and Findings:**

Thirty-two male HH patients with anosmia/hyposmia (Kallmann Syndrome, KS; n = 26) or normal sense of smell (nHH; n = 6) were enrolled (age range, 18–61 yrs). The patients were clinically examined, and reversal of HH was assessed after treatment withdrawal. *KAL1*, *FGFR1*, *FGF8*, *PROK2*, *PROKR2*, *CHD7*, *WDR11*, *GNRHR*, *GNRH1*, *KISS1R*, *KISS1*, *TAC3*, *TACR3*, and *LHβ* were screened for mutations. Six HH patients (2 KS, 4 nHH) were verified to have reversal of HH. In the majority of cases, reversal occurred early in adulthood (median age, 23 yrs; range, 21–39 yrs). All had spontaneous testicular growth while on testosterone replacement therapy (TRT). One nHH subject was restarted on TRT due to a decline in serum T. Two reversal variants had a same *GNRHR* mutation (R262Q), which was accompanied by another *GNRHR* mutation (R139H or del309F). In addition, both of the KS patients had a mutation in *CHD7* (p.Q51X) or *FGFR1* (c.91+2T>A).

**Conclusions:**

Considerable proportion of patients with HH (8% of KS probands) may recover in early adulthood. Spontaneous testicular enlargement during TRT was highly suggestive for reversal of HH. Those with the *GNRHR* mutation R262Q accompanied by another *GNRHR* mutation may be prone to reversal, although even patients with a truncating mutation in *CHD7* or a splice-site mutation in *FGFR1* can recover. We recommend that all adolescents and young adults with congenital HH should be informed on the possibility of reversal.

## Introduction

Delayed puberty predisposes adolescent to a significant psychosocial stress. Although constitutional delay of growth and puberty is the most common cause for pubertal delay, a small proportion of adolescents referred to evaluation for absent or delayed puberty has congenital hypogonadotropic hypogonadism (HH). Congenital HH is caused by the lack or deficient number of hypothalamic gonadotropin-releasing hormone (GnRH) neurons, disturbed secretion or action of GnRH, or both [1]–[4]. HH may occur with anosmia/hyposmia (Kallmann Syndrome, KS), or without it (normosmic HH, nHH). To induce pubertal development and to maintain adult sex steroid levels, the majority of HH male patients need life-long testosterone replacement therapy (TRT) [5]. However, up to 10% of HH patients may undergo reversal of hypogonadotropism and some of them even attain normal sperm count [6]. The phenotypic or genotypic features that would predict reversal are currently not known.

Although several cases with reversal of HH have been described [6]–[21], the clinical and molecular genetic features of these patients, and the triggers leading to reversal of HH are not well understood. For example, reversal variants have heterogeneous genetic background and may harbor mutation(s) in *KAL1* [18], *FGFR1* [16], [20], *PROK2* [19], *GNRHR* [15], [17], and *TAC3/TACR3* [21]. Age at reversal varies from 17 to 39 years, and the phenotype of younger patients may mimic delayed puberty [17]. Androgen exposure has been suggested to predispose to reversal [6], [22], although rare patients with a confirmed genetic cause for HH have been reported to recover from hypogonadotropism spontaneously [15], [23]. Thus, relatively little is known about the circumstances that predispose to reversal of HH.

Herein, we investigate whether Finnish reversal variants displayed a common phenotypic or genotypic feature that would predict the clinical course of HH.

## Methods

### Patients

Thirty-two male patients, previously diagnosed with KS or normosmic HH, who participated in the current study, were enrolled from the 5 different university hospitals in Finland. Patients were identified from hospitals' discharge registers by the International Code for Diseases edition 10 and 9 codes for hypogonadotropic hypogonadism (E23.04 and 253.4, respectively) and were invited to participate in our study if they fulfilled the following criteria: 1) absent or incomplete puberty by the age of 18, 2) low testosterone levels in association with normal or subnormal gonadotropin levels, 3) otherwise normal anterior pituitary function, and 4) no organic cause for their condition. We have previously reported the molecular genetic diagnoses of KS patients, except for proband #2 in the current study (see below) (24). All patients had received hormonal treatment to induce or complete pubertal development.

### Medical history, clinical examination, and assessment of HH reversal

Medical records were reviewed and patients were interviewed concerning their prior pubertal development and treatments (including treatment pauses), and prior biochemical measurements. Participants underwent physical examination as described [24]. Testicular volume was measured with a ruler (length × width^2^ ×0.52). Olfaction was tested by 40-item UPSIT test (University of Pennsylvania Smell Identification Test, Sensonic Inc, Haddon Heights, NJ), and defective ability to smell was defined by an UPSIT score <5^th^ percentile of age and/or absent or rudimentary olfactory bulbs in MRI [25].

HH reversal was assessed either prospectively based on treatment withdrawal, or retrospectively (if a subject had been off treatment) based on medical records and anamnestic information. In the current study, seven patients agreed to discontinue their hormone therapy to assess reversal prospectively. Reversal was diagnosed if they had normal serum reproductive hormone levels after an appropriate treatment washout period (1 mo for hCG injections, 3 mo for transdermal T, 3 to 6 mo for T injections), and no symptoms of hypogonadism after cessation of treatment. Those who did not fulfill these criteria were restarted on TRT. Three subjects had undergone reversal already before participating in this study. This retrospective identification of HH reversal was based on: 1) spontaneous testicular growth during and/or after TRT, 2) normal serum reproductive hormone levels and 3) no symptoms of hypogonadism after discontinuation of TRT.

### Biochemical measurements

Serum LH and FSH levels measured before and/or after the cessation of the hormone therapy were quantified with time-resolved immunofluorometric assays (AutoDELFIA, Wallac, Turku, Finland). The detection limit of the LH assay was 0.05 IU/L, and the interassay coefficient of variation (CV) was less than 4% in the concentration range 0.3–42 IU/L. For FSH, the detection limit was 0.05 IU/L, and the interassay CV was 5% or less in the concentration range 2–78 IU/L. Serum testosterone concentrations were measured with an API 2000 tandem mass spectrometer (AB Sciex, Foster City, California, USA) with a limit of detection 0.05 nmol/L. Interassay CVs were 4.2–7.6 % at mean concentrations of T of 3.3–45 nmol/L. Serum LH, FSH, and T values at the time of the diagnosis were obtained from medical records.

### Mutation screening

KS patients were screened for mutations in genes *KAL1*, *FGFR1*, *FGF8*, *PROK2, PROKR2, CHD7*, and *WDR11* as described [24]. Mutations in these genes have previously shown to cause HH together with anosmia. On the other hand, mutations in *FGFR1*, *FGF8*, *PROK2*, *PROKR2*, *CHD7*, and *WDR11* can also cause HH without an olfactory defect and were therefore screened in patients with normosmic HH. Normosmic patients were also screened for mutations in *GNRHR*, *GNRH1*, *KISS1R*, *KISS1*, *TAC3*, and *TACR3*, which are genes that only regulate secretion and/or action of GnRH from hypothalamus or pituitary without interfering development of olfactory track. In addition, normosmic subjects were screened for mutations in *LHβ*, in which a homozygous deletion in exon 2 was previously described in a male patient with a fertile eunuch variant of HH [26]. The coding exons and exon-intron boundaries of these genes were PCR-amplified, the PCR products were purified with ExoSAP-IT treatment (Amersham Biosciences, Piscataway, NJ, USA), and bi-directly sequenced using the ABI BigDyeTerminator Cycle Sequencing Kit (v3.1) and ABI Prism 3730xl DNA Analyzer automated sequencer (Applied Biosystems, Foster City, CA, USA). The sequences were aligned and read with Sequencher® 4.9 software (Gene Codes Corporation, Ann Arbor, MI, USA). All primer sequences and PCR conditions are available upon request.

### Ethics

The Ethics Committee of the Helsinki University Central Hospital approved the study protocol, appropriate permissions were provided from each university hospital in Finland, and all subjects signed the written informed consent.

### Statistical analysis

Statistical analyses were performed using IBM SPSS software version 19.0 (SPSS Inc. software, Chicago, Illinois, USA). Fisher's exact test was utilized to compare categorical variables between two groups, and two-sided *P*<0.05 was accepted to indicate statistical significance.

## Results

**Figure 1:**
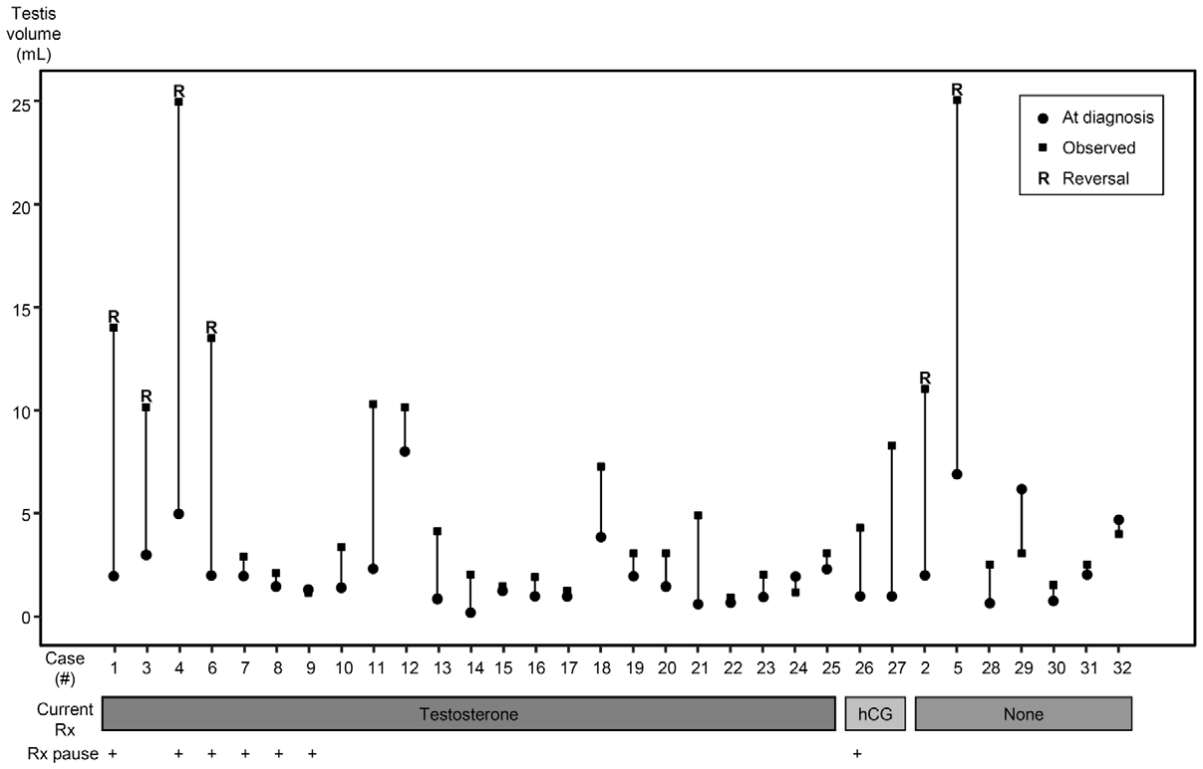
Assessment of the reversal of congenital hypogonadotropic hypogonadism (HH). Mean testicular volumes of HH patients were measured at the time of the diagnosis (solid circles) and at participation in the current study (small line). We used the value 2 mL for testicular volumes for cases #2, #6, #7, and #19 at diagnosis, because volumes were estimated to be “small" or “prepubertal" in medical records. Cases #21–25 were previously treated with recombinant-FSH and hCG to induce spermatogenesis. Seven patients (cases #1, 4, 6–9, and 26) discontinued hormone therapy, and 6 (cases #1–6) showed reversal of HH (4 during the treatment pause and 2 without TRT). In addition, #11, 13, 18, and 21 had spontaneous testis growth but they refused to discontinue TRT.

**Figure 2:**
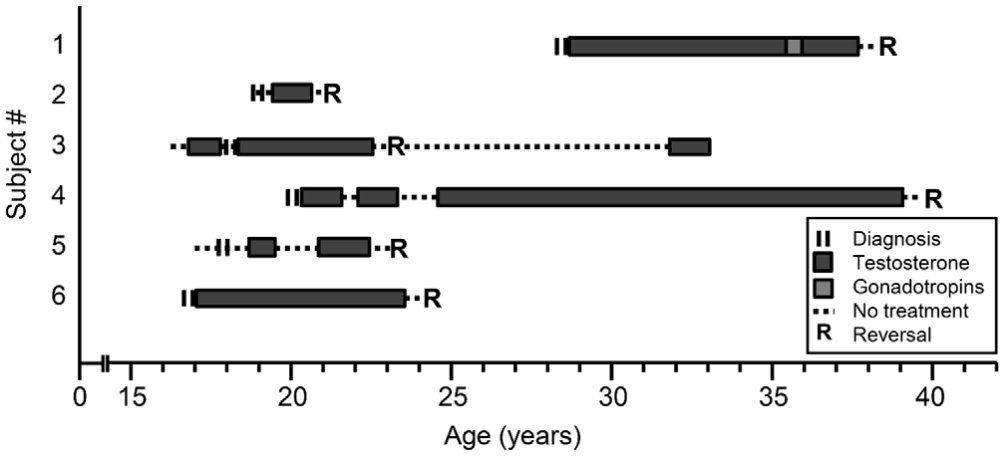
Schematic depicting time of diagnosis, treatment history, and reversal in 6 men with congenital hypogonadotropic hypogonadism.

We investigated the phenotypic and genotypic features of Finnish HH patients who underwent reversal of hypogonadotropism. Among the 32 probands (26 KS, 6 nHH) enrolled, seven were observed to have had spontaneous testicular growth while on TRT (2 KS and 3 nHH patients) or while off hormone therapy (1 KS and 1 nHH) (Fig. 1). Furthermore, four patients (#11, 13, 18, and 21 in Fig. 1) had enlarged testes; however, they did not want to cease TRT to further assess the reversal of HH. Three patients without spontaneous testis growth (#7, #8, and #9; Fig.1) and one who had received hCG (#26; Fig. 1), also had treatment pauses (range, 2 to 6 months) but they did not display evidence for reversal, and were therefore restarted on TRT due to symptoms of hypogonadism and low serum reproductive hormone values. Therefore six probands (2 KS and 4 nHH) were confirmed to undergo reversal of HH, of which three were identified prospectively after treatment withdrawal and three retrospectively. Treatment histories and testicular volumes, sex hormone and gonadotropin levels at the diagnosis of HH and after TRT pause in these 6 probands with HH reversal are summarized in Figure 2 and Table 1. It is noteworthy that these 6 patients were exposed to androgens before reversal (Fig. 2). Thus, the frequency estimate of HH reversal among KS patients was 8% (2/26). Although frequency estimates of HH reversal differed significantly between KS and nHH (4/6; 67%) subjects (*P* = 0.006), the frequency of nHH reversals might be overestimated due to small number of patients.

Among the 6 probands with verified reversal, the median age at reversal of HH was 23 yrs, ranging from 21 to 39 yrs. None of the probands with reversal had been treated for cryptorchidism or micropenis in infancy. Detailed case histories of these patients are described in supplement data (Text S1). Three men who were prospectively identified as reversal variants (#1, #4, #6) (age, 24–39 yrs) have had spontaneous testicular growth during TRT, and, after cessation of the treatment for 6–12 months, maintained normal T and gonadotropin levels (Table 1). It should be noted, however, that the testicular enlargement in one patient on hCG indicated treatment effect and not reversal of HH. Sperm analysis of subject #1 showed normal sperm count (35×10^6^/mL). Two patients with normosmic HH (#3 and #5) and one patient with KS (#2) had had reversal already before participating in the current study: They had been off treatment for several years, and, during that time, had had no symptoms of hypogonadism. However, subject #3 had begun to suffer from fatigue and decreased libido, and he had low serum T (1.9 nmol/L), LH (1.5 IU/L) and FSH (0.8 IU/L) levels. Therefore, he was restarted on TRT after 9 years of reversal (Fig. 2). Overall, three probands had fathered a child during TRT (#1 and #4) or without it (#2).

**Table 1:** Clinical and biochemical features of patients with congenital hypogonadotropic hypogonadism (HH) before and after reversal of HH.

			At diagnosis					After reversal of HH					
Subject	Diagnosis	Mutated Gene	Age	T (nM)	LH (IU/L)	FSH (IU/L)	Tvol (mL)	Age	Time without Rx	T (nM)	LH (IU/L)	FSH (IU/L)	Tvol (mL)
1	KS	*CHD7*	28	2.8	0.9	0.5	2	38	12 mo	12.0	2.6	2.8	16
2	KS	*FGFR1*	19	2.7	1.7	1.5	“Small"	36	15 yrs	10.7	3.9	3.1	11
3	nHH	*GNRHR*	18	1.4	0.8	0.9	5	22	3 mo	19.3	4.4	3.9	“Normal"
4	nHH	*GNRHR*	20	1.3	0.2		5	39	9 mo	13.6	3.9	2.9	25
5	nHH		18	1.7	2.9	0.5	7	38	16 yrs	19.0	3.8	2.3	25
6	nHH		17	1.7	0.1	0.6	“Small"	24	6 mo	10.1	3.3	4.1	14

Tvol, testicular volume; KS, Kallmann Syndrome; nHH, normosmic HH. Normal adult men reference range for testosterone (T) 10–38 nmol/L, for luteinizing hormone (LH) 1.7-8.6 IU/L, and for follicle-stimulating hormone (FSH) 1.5-12.4 IU/L.

### Mutation screening in patients with reversal of HH

We have previously described detailed molecular genetic features of the patients with KS [24]. The two KS probands with reversal of HH both obtained molecular genetic diagnosis: proband #1 from a previous study harbored a heterozygous truncating mutation in *CHD7*, c.151C>T (p.Q51X) [24], and the proband #2 had a heterozygous mutation in the intron 2 of *FGFR1*, c.91+2T>A that destroys the conserved donor splice site consensus sequence of exon 2 (http://www.cbs.dtu.dk/services/NetGene2/). Of note, none of the three men with *KAL1* mutation [24] had features suggesting reversal of HH, whereas at least 1 out of 6 (17%) of KS male patients with an *FGFR1* mutation reversed [24]. A high proportion of nHH subjects harbored compound heterozygous mutations in *GNRHR*. The proband #3 had *GNRHR* mutations c.416G>A (p.R139H) and c.785G>A (p.R262Q) that both have been previously described in patients with HH (27–29). His father and mother were unaffected heterozygous carriers of the respective mutations. Proband #4 harbored the R262Q mutation together with a c.924_926delCTT (p.del309F) mutation absent from 200 controls [27]. His unaffected mother and father were heterozygous carriers of the respective mutations. We did not find mutations in the two remaining patients (#5 and #6) with reversal of HH.

## Discussion

We studied phenotypic and genotypic features of the patients who have undergone reversal of congenital HH. In most cases the reversal of HH took place soon after transition from pediatric to adult healthcare, and thus, adolescent patients with congenital HH should be thoroughly informed on the possibility of endogenous puberty and spontaneous fertility in adulthood especially in patients with normosmic HH. Indeed, our frequency estimate for the reversal of HH among KS patients was 8%, which is in accordance with the 10% reported in the US among patients with congenital HH [6].

Consistent with previous reports [6], [7], [13], four subjects (#2, 3, 5, and #6) displayed reversal before the age of 25, and remained eugonadal several years after cessation of the treatment. It may be argued that their clinical presentation resembled extreme constitutional delay of puberty (CDP). However, nHH subjects' testosterone exposure had lasted for years before reversal, proband #3 was later restarted on TRT due to a decline in serum T, and the proband #2 presented with unilateral olfactory bulb aplasia. Neither these clinical features, however, nor the fact that HH diagnosis was confirmed by molecular genetic analyses in two thirds of reversal patients, are consistent with CDP.

Two men with reversal of HH had fathered a child while on TRT and one became a father when off Rx. Similar case reports have been described previously [8], [12], [14]. Based on the data in normal men [28], exogenous T should suppress rather than initiate spermatogenesis. It is therefore tempting to speculate that, following reversal of congenital HH, gonadotropin secretion is less sensitive to exogenous T. This discrepancy may also result from differences in T dosing when given for replacement therapy or for male contraception.

Although we did not find common clinical features among reversal variants that would have predicted the clinical course of HH after adolescence, two reversal variants had mutations in *GNRHR*. Once these data are added to the previous data on HH reversals [6], [15]–[17], [20], [29], *GNRHR* mutations are one of the leading molecular genetic diagnoses among reversal cases. Especially, two common *GNRHR* mutations, R262Q and Q106R, have previously been associated with mild phenotypes [15], [17], [29], [30]. Our two patients both had an R262Q mutation (compound heterozygotes R139H/R262Q and R262Q/del309F). Therefore, it could be possible that HH patients with an R262Q mutation in *GNRHR* are especially prone to reversal of HH.

For the first time, we demonstrate that a KS patient with a truncating *CHD7* mutation can undergo reversal of hypogonadotropism. A *CHD7* mutation is found in >70% of patients with CHARGE syndrome (coloboma, heart defect, atresia choanae, retarded growth and development, genital hypoplasia, ear anomalies/deafness) [31] and in ∼5% of KS patients [32], [33]. The latter are suggested to represent a mild end of CHARGE phenotypic spectrum due to overlapping features of these syndromes such as hypogonadism, anosmia, cleft lip and palate, hearing impairment, and semicircular canal hypoplasia [32], [33]. The relatively mild phenotype of our patient with the truncating mutation Q51X in *CHD7* [24] demonstrates that nonsense mutations are not always fully penetrant for CHARGE syndrome [32]. A similar case was reported recently, where a girl with a *CHD7* mutation and had delayed pubertal development [34]. However, due to young age of this patient, it remained unsure whether or not she entered puberty later in life. Anosmia in CHARGE patients is suggested to predict the presence of HH [35], [36]. On the other hand, Feret *et* *al.* described one anosmic male patient, with a *CHD7* mutation, who had apparently normal fertility [37]. This phenotypic variability may be inherent to effect(s) of modifier genes, and/or environmental factors. Alternatively, *CHD7* is one of the key genes for the formation of cranial neural crest [38], which has been suggested to be the source of ∼30% of GnRH neurons in mice [39]. Thus, it is possible, that our patient had reduced (but not absent) GnRH cell number due to disrupted development of neural crest, defective formation of the olfactory system and subsequent misrouting of GnRH neurons, or both. Interestingly, *Chd7* haploinsufficiency in mice leads to decreased expression of *Fgfr1* during development, as well as reduced *Gnrh1* in the adult hypothalamus [40], suggesting that proper CHD7 signaling is required both for normal GnRH neurogenesis and GnRH signaling.

In conclusion, reversal of congenital HH is a relatively common feature that typically takes place in early adulthood. Spontaneous testicular growth during androgen therapy is highly indicative for reversal of HH. Normosmic HH patients with a R262Q mutation in *GHRHR* accompanied by other *GNRHR* mutation may be prone to reversal of HH. We also show that patients with a truncating *CHD7* mutation or a splice-site mutation in *FGFR1* can undergo reversal. We recommend that all patients with congenital HH should be informed by pediatricians before transition to adult healthcare on the possibility of HH reversal.

## Supporting Information

Text S1 **Reversal case histories.**
(DOC)
