# Supplementary material for: Reversible Congenital Hypogonadotropic Hypogonadism in Patients with CHD7, FGFR1 or GNRHR Mutations
Source: PLoS One. 2012 Jun 19;7(6):e39450. doi: 10.1371/journal.pone.0039450 (PMC3378565; doi:10.1371/journal.pone.0039450)
Supplement: Text S1 — Reversal case histories. (DOC) [file pone.0039450.s001.doc]

###### Text S1. Reversal case histories

*Subject #1* (24) was referred to endocrinologist relatively late, at the age of 28. He had undergone only partial puberty, and had high-pitched voice, no facial hair, and decreased sense of smell. He has no family history of delayed puberty, KS, HH, or anosmia. Laboratory tests showed low T and gonadotropin levels (Table 1), and therefore he was diagnosed with KS and started on TRT. He had some spontaneous testicular growth during the treatment (ad 12- 15 ml), and to induce spermatogenesis, testosterone was replaced by combined gonadotropin treatments seven yrs after the diagnosis. Only after few months of gonadotropin treatment, his wife got pregnant, and TRT was continued. After he fathered his second child during TRT, the treatment was discontinued. During the 12-month follow-up, he has maintained normal levels of T and gonadotropins, had no symptoms of hypogonadism, and sperm analysis showed normal sperm count (35 x 10^6^/mL). His additional phenotypic features include also mild hearing loss and semicircular canal hypoplasia of the inner ear. Molecular genetic analysis revealed a truncating nonsense mutation c.151C>T (p.Q51X) in *CHD7* (24).

*Subject #2* was referred to endocrinologist at the age of 19 due to delayed pubertal development. He had no family history of KS, nHH, or anosmia, but his father was reported to have delayed puberty. At birth the proband had unilateral external ear anomaly, but no signs of hypogonadism. At the age of 19 he was found to have decreased sense of smell (hyposmia), and in physical examination he had eunukoid habitus and small testis, and therefore he was start on T to induce puberty. After one year of TRT, he had undergone puberty and had had spontaneous testicular growth, but he was lost for follow-up. At the age of 36 he participated in this study, and reported that he had discontinued the TRT at the age of 21, had no symptoms of hypogonadism thereafter, and had fathered two children without any hormonal treatment. In clinical examination, he had normal testis volumes (10 and 12 mL), eugonadal habitus, and normal serum reproductive hormone levels (T 10.7 nmol/L; LH 3.9 IU/L; FSH 3.1 IU/L) (Table 1). MRI showed unilateral olfactory bulb aplasia. He had a mutation in the intron 2 of *FGFR1*, c.91+2T>A that destroys the conserved donor splice site consensus sequence of exon 2 (http://www.cbs.dtu.dk/services/NetGene2/).

*Subject #3* was followed-up from the age of 16 due to absent pubertal development. His brother, mother, and father all had normal onset of puberty, but the father was reported to have slightly delayed growth spurt. Also, the proband has normal sense of smell and had no cues for HH at birth. He was started on T to induce puberty at the age of 17. The treatment was discontinued at the age of 18 for 7 months. By that time his Tanner stage was P4 G4, and there was no improvement in puberty during the cessation of the treatment. Before restarting the TRT, his serum T level was 1.4 nmol/l, and GnRH stimulation test showed normal LH response (0.9 – 12.0 IU/L), but low FSH response (0.9 – 1.9 IU/L). At the age of 22, he had completed pubertal development and after the cessation of the treatment he maintained normal sex hormone and gonadotropin levels (Table 1). However, during the years without hormonal treatment he began to suffer from symptoms of hypogonadism (fatigue, decreased libido), his serum hormone levels were low (T 1.9 nmol/L; LH 1.5 IU/L; FSH 0.8 IU/L), and the treatment was restarted after nine years of treatment pause at the age of 31. The subject was found to harbor two *GNRHR* missense mutations, c.416G>A (p.R139H) and c.785G>A (p.R262Q) in a compound heterozygous state. His father and mother were unaffected heterozygous carriers of the mutations (R139H and R262Q, respectively).

*Subject #4* was evaluated at the age of 20 due to incomplete pubertal development. He has normal sense of smell, and his father was reported to have delayed growth spurt. There was no family history of HH, KS, or anosmia, and his mother and siblings had normal onset of puberty. He was diagnosed with normosmic HH (Table 1) and the puberty was induced with T. During the treatment he had spontaneous testicular growth, and his treatment was discontinued two times (for 6 and 12 months). However, after both treatment pauses his serum T level remained very low, and TRT was continued. Sixteen years after the diagnosis, while still on TRT, he fathered a child. Two years later, TRT was discontinued, and, during 9-months of follow-up, he had no symptoms of hypogonadism and biochemical testing showed normal serum hormonal values (Table 1). Mutation analysis revealed compound heterozygous *GNRHR* mutations, c.785G>A (p.R262Q) and c.924_926delCTT (p.del309F). His father and mother were unaffected heterozygous carriers of the mutations (del309F and R262Q, respectively).

*Subject #5* was followed-up for delayed puberty from the age of 17. He had no family history of HH or associated features, and has normal sense of smell. Because he had not completed pubertal development by the age of 18, and laboratory tests showed low basal T, LH and FSH values (Table 1), he was started on T at the age of 19. He had spontaneous testicular growth during the treatment (ad 17 mL), but after six months cessation of TRT the biochemical testing still showed HH, and therefore TRT was continued. At the age of 23 he had completed pubertal development and maintained normal serum hormone values after cessation of treatment. Since then, he has been followed-up for symptoms of HH, but he has remained eugonadal and had normal serum hormone values (Table 1). No mutations were found in the genes analyzed.

*Subject #6* was referred to endocrinologist due to absent pubertal development at the age of 17. He has normal sense of smell, no history of cryptorchidism or micropenis, and no family history of KS, HH, or delayed puberty. He also suffered from severe obesity (BMI 47), but laboratory tests showed no abnormality in leptin or cortisol secretion, or in thyroid function. Because he had low basal gonadotropin levels combined with low T level, but GnRH stimulation test showed good response (LH <0.1 – 64.3; FSH 0.6 – 24.5 IU/l), he was started on T therapy to induce pubertal development at the age of 17. Few years after the onset of treatment, he was lost to follow-up. At the age of 24 he participated in our study, and he had no TRT for 6 months before participating. In physical examination, he had normal testicular volumes and masculine habitus, he did not suffer from symptoms of hypogonadism, and had normal serum reproductive hormone values (Table 1). No mutations were found in the genes analyzed.
